# Genome Sequencing Analysis of *Scleromitrula shiraiana*, a Causal Agent of Mulberry Sclerotial Disease With Narrow Host Range

**DOI:** 10.3389/fmicb.2020.603927

**Published:** 2021-01-14

**Authors:** Zhiyuan Lv, Ziwen He, Lijuan Hao, Xin Kang, Bi Ma, Hongshun Li, Yiwei Luo, Jianglian Yuan, Ningjia He

**Affiliations:** State Key Laboratory of Silkworm Genome Biology, Southwest University, Chongqing, China

**Keywords:** mulberry sclerotial disease, fungal genomics, necrotrophic fungus, secondary metabolism, cell wall-degrading enzymes, effector

## Abstract

*Scleromitrula shiraiana* is a necrotrophic fungus with a narrow host range, and is one of the main causal pathogens of mulberry sclerotial disease. However, its molecular mechanisms and pathogenesis are unclear. Here, we report a 39.0 Mb high-quality genome sequence for *S. shiraiana* strain SX-001. The *S. shiraiana* genome contains 11,327 protein-coding genes. The number of genes and genome size of *S. shiraiana* are similar to most other Ascomycetes. The cross-similarities and differences of *S. shiraiana* with the closely related *Sclerotinia sclerotiorum* and *Botrytis cinerea* indicated that *S. shiraiana* differentiated earlier from their common ancestor. A comparative genomic analysis showed that *S. shiraiana* has fewer genes encoding cell wall-degrading enzymes (CWDEs) and effector proteins than that of *S. sclerotiorum* and *B. cinerea*, as well as many other Ascomycetes. This is probably a key factor in the weaker aggressiveness of *S. shiraiana* to other plants. *S. shiraiana* has many species-specific genes encoding secondary metabolism core enzymes. The diversity of secondary metabolites may be related to the adaptation of these pathogens to specific ecological niches. However, melanin and oxalic acid are conserved metabolites among many *Sclerotiniaceae* fungi, and may be essential for survival and infection. Our results provide insights into the narrow host range of *S. shiraiana* and its adaptation to mulberries.

## Introduction

Mulberry (*Morus* spp.) is the feed crop for silkworms (*Bombyx mori* L.). In addition, mulberry fruit also has great economic and medicinal value because it is rich in secondary metabolites that are beneficial to human health (Chen et al., 2016, 2017; Choi et al., 2016). Mulberry sclerotial disease is the most serious fungal disease of *Morus* spp., and it severely reduces fruit yield. However, effective prevention and control measures are lacking. Three fungi in the *Sclerotiniaceae*, *Ciboria carunculoides*, *Ciboria shiraiana*, and *Scleromitrula shiraiana*, cause mulberry sclerotial disease (Whetzel and Wolf, 1945; Hong et al., 2007). *S. shiraiana* is widely distributed in major mulberry cultivation areas in East Asia, such as China, South Korea, and Japan (Schumacher and Holst-Jensen, 1997). *S. shiraiana* has a similar life cycle to that of *C. carunculoides* and *C. shiraiana*, and forms sclerotia in infected drupelets. Ascospores, which are produced by the fruiting bodies that germinate from the sclerotia, are the source of primary infection.

**FIGURE 1:**
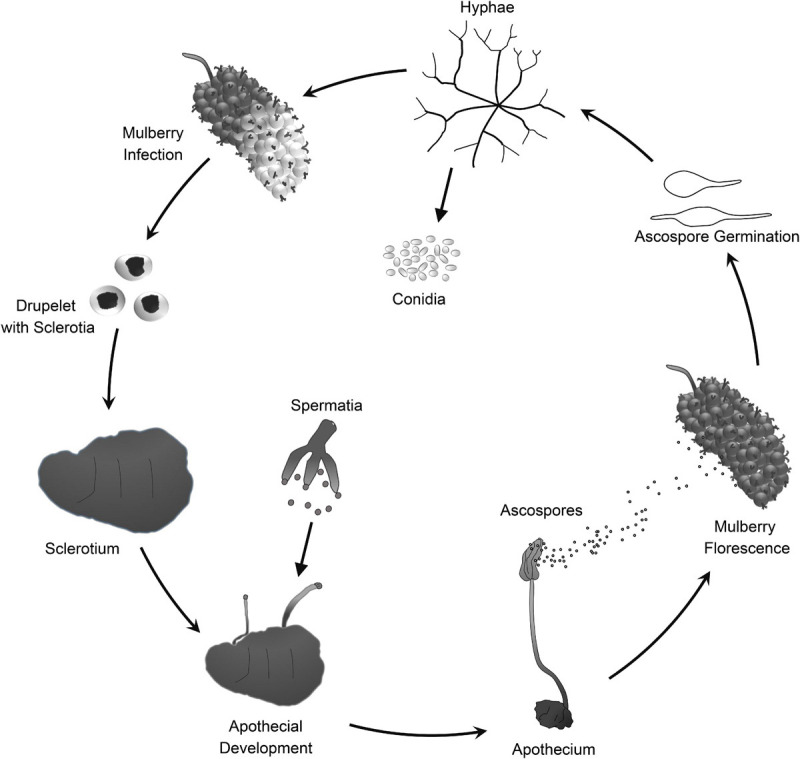
Lifecycle of *Scleromitrula shiraiana.*

*Scleromitrula shiraiana* is a typical necrotrophic plant pathogenic fungus, which is phylogenetically closely related to the notorious *Sclerotinia sclerotiorum* and *Botrytis cinerea*. Their life cycles are also similar, and all of them belong to the Sclerotinaceae family (Ascomycota) (Figure 1). Both *S. sclerotiorum* and *B. cinerea* have considerably broader host ranges than that of *S. shiraiana*, causing diseases in more than 400 and 1000 plant species, respectively, including many important crops (Bolton et al., 2006; Williamson et al., 2007; Elad et al., 2016). However, *S. shiraiana* causes disease only in mulberry. *S. shiraiana* can produce conidia by asexual propagation like *B. cinerea*, but different from *S. sclerotiorum*. However, in *S. shiraiana*, the role of conidia in the infection cycle is insignificant, because the time window suitable for pathogen infection is narrow. In addition, conidia of *S. shiraiana* are formed in a liquid environment, which is not conducive to spreading. Another obvious difference between *S. shiraiana* and its relatives *S. sclerotiorum* and *B. cinerea* is that *S. shiraiana* shows is very slow growth on potato dextrose agar or other artificial media. Sexual reproduction is essential for the inheritance and variation of fungi. *S. sclerotiorum* is self-fertilized and homothallic. However, the sexual process of most *B. cinerea* strains is heterothallic, i.e., it needs two different mating-type strains. The different mating types of Ascomycetes are determined by the mating-type (*MAT*) gene at the *MAT* locus. The transcription factors encoded by the *MAT* gene are required for sexual reproduction (Debuchy et al., 2010). Homothallic *S. sclerotiorum* has two types of *MAT* genes at one *MAT* locus, namely *MAT1-1* and *MAT1-2* (Malvárez et al., 2007). However, like heterothallic *B. cinerea*, different idiomorphs of *S. shiraiana* are in two types of strains.

Necrotrophic plant pathogens extract nutrients from dead cells that are killed prior to or during colonization. Phytotoxic compounds and cell wall-degrading enzymes (CWDEs) are common attack weapons of necrotrophs, and are deployed to induce plant cell necrosis and cause leakage of nutrients (Mengiste, 2012). Many CWDEs contribute to the pathogenicity of *S. sclerotiorum* and *B. cinerea* (Riou et al., 1991; Alghisi and Favaron, 1995; Cotton et al., 2003; Kars et al., 2005; Brito et al., 2006). The decrease in the production of numerous CWDEs was associated with a concomitant decrease in virulence of pathogens (Kubicek et al., 2014). Genomic analyses have revealed abundant genes encoding CWDEs in the genomes of *S. sclerotiorum* and *B. cinerea* (Amselem et al., 2011; Derbyshire et al., 2017; Van Kan et al., 2017). For many necrotrophs, CWDEs are their common weapons, while many phytotoxins are specific to particular pathogens. Botrydial and botcinic acid are two types of phytotoxins produced by *B. cinerea* (Colmenares et al., 2002; Tani et al., 2006). Several relevant biosynthetic genes have been identified, such as the polyketide synthase gene *BcPKS6* and *BcPKS9* (also known as *Bcboa6* and *Bcboa9*), and *BcBOT1* to *BcBOT5*, respectively (Dalmais et al., 2011; Collado and Viaud, 2016). Another phytotoxin, oxalic acid, is a crucial virulence factor shared by *S. sclerotiorum* and *B. cinerea*. Oxalic acid can promote virulence in several ways: It enhances the activity of polygalacturonases to promote cell wall degradation, suppresses the plant oxidative burst, promotes programmed cell death (PCD) in the host, and alters the redox in plant cells (Cessna et al., 2000; Favaron et al., 2004; Kim et al., 2008; Williams et al., 2011). Oxalic acid has also been detected in *S. shiraiana*, and may contribute to its pathogenicity during infection of mulberry fruits. Furthermore, there is a positive correlation between oxalic acid and melanin contents in *S. shiraiana* (Lü et al., 2017). Melanin plays roles in the self-protection and pathogenicity of pathogens (Butler and Day, 1998; Henson et al., 1999; Jacobson, 2000; Butler et al., 2001). Pathogens with melanized appressoria have stronger pathogenicity (Ebbole, 2007; Butler et al., 2009). For sclerotia-forming fungi, such as *S. sclerotiorum*, *B. cinerea*, and *S. shiraiana*, melanin is an essential component of the sclerotia (Butler et al., 2009; Liang et al., 2018). The genes involved in the biosynthesis of 1,8-dihydroxynaphthalene melanin are conserved in corresponding fungi.

A growing body of evidence suggests that the effector proteins of necrotrophic fungi suppress the plant immune response and modulate plant physiology to accommodate fungal invaders (Presti et al., 2015). Since the interaction between the necrotrophic effectors and their hosts operate opposite of the classic gene-for-gene interaction observed in host-biotroph systems, it is called inverse gene-gene interaction (Faris and Friesen, 2020). Cerato-platanins (CP) are a group of small, secreted, cysteine-rich proteins that have been found only in fungi (Pazzagli et al., 2014). Most CP proteins have been shown to act either as virulence factors or as elicitors. However, in necrotrophic fungi, the CP protein is related to fungal virulence. A previous study showed that BcSpl1 is required for virulence in *B. cinerea* and promotes pathogenic infection by inducing hypersensitive cell death in the host (Marcos et al., 2011). SsSm1 and SsCP1 are two members of the CP family in *S. sclerotiorum*. Transient expression of *SsSm1* and *SsCP1* in tobacco leaves was found to cause a hypersensitive response (Pan et al., 2018; Yang et al., 2018). SsCP1 was shown to interact directly with the pathogenesis-related protein PR1 in the apoplast, thereby inhibiting its potential antifungal activity. However, the functions of most fungal effector proteins are unknown, and they lack conserved domains or homologs in other fungi.

It is noteworthy that both BcSpl1 and SsCP1 induce an increase in the salicylic acid (SA) concentration in the host (Marcos et al., 2013; Yang et al., 2018). In plants, SA is required to activate defenses against biotrophic and hemi-biotrophic pathogens, and it is essential in local resistance and systemic acquired resistance (SAR) (Qi et al., 2018). Local resistance induces hypersensitive response (HR)-like cell death at the infected site, making the plant resistant to biotrophic and hemi-biotrophic pathogens, but susceptible to necrotrophic pathogens. However, SAR is broad-spectrum resistance to a variety of pathogens, including necrotrophs (Vlot et al., 2009). Thus, SA may be an ancient plant hormone involved in defense, but it may also play a more complex role in the arms race between plants and necrotrophic pathogens.

As mentioned above, *S. shiraiana* is a pathogen with a narrow host spectrum unlike *S. sclerotiorum* and *B. cinerea*. High-quality and almost complete genome sequences are available for *S. sclerotiorum* and *B. cinerea.* Previous studies have also reported the genomes of some obligate parasitic fungi and restricted host range necrotrophs such as powdery mildew fungi, rust fungi, *Parastagonospora nodorum*, and *Pyrenophora teres* f. *teres* (Hane et al., 2007; Ellwood et al., 2010; Spanu et al., 2010; Duplessis et al., 2011; Zheng et al., 2013; Schwessinger et al., 2018). Genomic analyses of pathogenic fungi with different nutritional styles can provide clues about the reasons for the differences in their host ranges.

In this study, we sequenced the genome of *S. shiraiana*, which is only known to infect mulberry. Our results show that the number of genes and genome size of *S. shiraiana* are similar to those of other necrotrophic phytopathogenic fungi. Effector proteins and CWDEs are common offensive weapons of necrotrophic pathogens. However, *S. shiraiana* has fewer genes encoding effectors and CWDEs than do *S. sclerotiorum* and *B. cinerea*, even though they are taxonomically closely related. Many phytotoxins of pathogens are secondary metabolites, and we detected major differences in secondary metabolism genes among *S. shiraiana*, *S. sclerotiorum*, and *B. cinerea*. This suggests that *S. shiraiana* may produce specific toxins to promote infection. In summary, the reduced number of key pathogenicity-related genes and the differences in secondary metabolism genes may jointly contribute to the adaptation of *S. shiraiana* to mulberry.

## Materials and Methods

### Strains, Culture Conditions, and Nucleic Acid Isolation

*Scleromitrula shiraiana* strain SX-001 was isolated from diseased mulberry fruit in our laboratory (Lü et al., 2017). The strain was routinely sub-cultured on potato dextrose agar (PDA) medium at 25°C to maintain vigor. Mycelia were collected from the media and ground in liquid nitrogen. Genomic DNA was extracted using a modified cetyltrimethylammonium bromide (CTAB) method (Umesha et al., 2016). Total RNA was extracted with TRIzol reagent (Invitrogen, Carlsbad, CA, United States) according to the manufacturer’s instructions and treated with RNase-free DNase I to remove contaminating DNA.

### Genome Sequencing and Assembly

The genome of *S. shiraiana* strain SX-001 was sequenced by Single Molecule Real-Time (SMRT) technology at the Beijing Novogene Bioinformatics Technology Co., Ltd. (Beijing, China). The DNA sample used for genome sequencing was assessed by Qubit Fluorometer and agarose gel electrophoresis. The 350-bp library was constructed following the standard protocols of Illumina (San Diego, CA, United States). The 350-bp library was quantified using an Agilent 2100 Bioanalyzer (Agilent, Palo Alto, CA, United States) and subjected to paired-ended 150-bp sequencing by Illumina HiSeq PE150. For long reads sequencing, 20-kb SMRTbell libraries were constructed following the standard methods of PacBio (Menlo Park, CA, United States). The 20-kb libraries were sequenced on a PacBio RSII instrument. The low quality reads were filtered by the SMRT Link v5.0.1 and the filtered reads were assembled to generate one contig without gaps. Finally, 21 high quality contigs were obtained.

### Genome Annotations

Protein-coding genes were annotated from the genome sequences using MAKER2 pipeline (Holt and Yandell, 2011). First, interspersed repetitive sequences were predicted by the RepeatMasker (Version open-4.0.5) combined with RepBase-20181026. Tandem Repeats were analyzed by TRF (Tandem repeats finder, Version 4.07b). Second, *ab initio* gene prediction was performed with protein-coding sequences from two strains in *B. cinerea* (B05.10 and T4), *S. sclerotiorum*, *S. borealis*, *Botrytis paeoniae*, and *Botrytis tulipae* (Amselem et al., 2011; Mardanov et al., 2014; Valero-Jiménez et al., 2019). For transcriptome sequences, we used a locally assembled transcriptome from RNA-Seq data using Trinity v2.11.0 (Grabherr et al., 2011). Third, two gene predictors were used in the pipeline: GeneMark-ES version 4.62 and Augustus version 3.3.3 (Ter-Hovhannisyan et al., 2008; Keller et al., 2011).

The following seven databases were used to predict gene functions: GO (Gene Ontology), KEGG (Kyoto Encyclopedia of Genes and Genomes), KOG (Clusters of Orthologous Groups), NR (Non-Redundant Protein Database databases), TCDB (Transporter Classification Database), P450, and, Swiss-Prot. A whole genome BLAST search (*E*-value < 1e-5, minimal alignment length percentage >40%) was also performed against the above seven databases.

### Comparative Analyses of Genes Encoding Carbohydrate-Active Enzymes and Secondary Metabolism Clusters and Genes

The identification and annotation of all 16 fungal carbohydrate-active enzymes were based on the Carbohydrate-active enzymes (CAZy) database and performed on the dbCAN2 meta server (Lombard et al., 2013; Zhang et al., 2018). All 16 fungal secondary metabolism clusters and genes were predicted by antiSMASH fungal version with default parameters (Blin et al., 2019). Genes encoding polyketide synthase (PKS), non-ribosomal peptide synthetase (NRPS), PKS-NRPS hybrid (HYBRID), terpene synthase (TS), and dimethylallyl tryptophan synthase (DMATS) were confirmed by NCBI BLASTP and CDD searches. The fungal genomes used for comparative analyses were downloaded from DOE Joint Genome Institute (JGI)^1^ and Ensembl Fungi^2^.

### Gene Family and Phylogenetic Analysis

Ortholog families in the *S. shiraiana* genome and 15 other fungal genomes were identified by OrthoFinder v2.4.0 (Emms and Kelly, 2019). The phylogenetic analysis was conducted using STAG (Species Tree Inference from All Genes) and STRIDE (Species Tree Root Inference from gene Duplication Events) methods, which infer a species tree from sets of multi-copy genes (Emms and Kelly, 2017, 2018).

### Melanin Synthesis Gene Cluster Analysis

Two *PKS* genes (sshi00009473 and sshi00001626), which are associated with 1,8-dihydroxynaphthalene (DHN) melanin synthesis in *S. shiraiana*, were identified based on homologous genes in *B. cinerea* and *S. sclerotiorum*. Genes encoding two other key enzymes for melanin synthesis, 1,3,6,8-tetrahydroxynaphthalene reductase (4HNR), and scytalone dehydratase (SCD), were identified by BLASTP in the genome of *B. cinerea* and *S. sclerotiorum*. *ShPKS13* (sshi00001626) is located in the same gene cluster as *ShSCD* (sshi00001628) and *Sh4HNR* (sshi00001627).

### Secretory Proteins and Putative Effectors

The secretory proteins were predicted using SignalP 5.0 (Almagro Armenteros et al., 2019). Then, TMHMM 2.0 was used to filter out those containing transmembrane domains (Krogh et al., 2001). Potential virulence-related proteins were identified by searching against the pathogen-host interaction database (PHI base) by BLASTP (Urban et al., 2016). To predict effectors, known secretory protein sequences were entered into the Big-PI Fungal Predictor to filter out proteins harboring a putative glycophosphatidylinositol membrane-anchoring domain (Eisenhaber et al., 2004). Finally, EffectorP 2.0 was used to predict potential effectors in the remaining proteins (Sperschneider et al., 2018).

### Transient Expression Analysis of Putative Effectors in *Nicotiana benthamiana*

The putative effector genes were cloned into pGR107 by homologous recombination with the pEASY Basic Seamless Cloning and Assembly Kit (TransGen, Beijing, China) according to the manufacturer instructions. The GR107 vector was linearized by digestion with *Cla*I and *Sal*I. Then, each construct was transformed into *Agrobacterium tumefaciens* strain GV3101 containing the helper plasmid pJIC SA_Rep. Infiltration experiments were performed on 4- to 6-weeks-old *N. benthamiana* plants using needleless syringes as described before (Lv et al., 2018). The negative control was GFP and the positive control was the pro-apoptotic mouse protein BAX. Cell death symptoms were photographed at 6 days after infiltration. The results are representative of three biological replicates. The primer sequences are listed in Supplementary Table 8.

### Obtaining and Analysis of *ShSCD-*Deletion Mutants

Deletion of *ShSCD* was performed as described previously by Yu et al. (2016). The gene deletion utilizes pSKH vector. The primer pair SCD3XhoIF and SCD3KpnIR were used to amplify a *c.* 800 bp sequence of 3′ untranslated region (UTR) of *ShSCD* gene. The fragment was digested with *Xho*I and *Kpn*I and then inserted into pSKH to produce the pSKHSCD1. The primer pair SCD5SacIF and SCD5NotIR were used to amplify a *c.* 800 bp sequence of 5′ UTR of *ShSCD* gene. The fragment was digested with *Sac*I and *Not*I and then inserted into pSKHSCD1 to produce the pSKHSCD2. The pSKHSCD2 plasmid was used as a PCR template, and the 5′UTR:N-HY and 3′UTR:C-YG fragments were amplified using primer pairs SCD5-F/HY-R and YG-F/SCD3-R, respectively. Then two fragments were transformed into protoplasts of wild-type *S. shiraiana* SX-001 strain as described by Rollins (2003). The *ShSCD-*deletion mutants were identified by PCR and Southern blot hybridization. For Southern blotting, probes were designed based on the gene sequence of *ShSCD* and the CDS of the gene encoding the hygromycin B phosphotransferase (HYG). The probes were labeled with the DIG High Prime DNA Labeling and Detection Starter Kit II (Roche, Indianapolis, IN, United States) according to the manufacturer’s instructions. Genomic DNA was extracted from fungal strains using the CTAB method (Umesha et al., 2016). Southern blotting was performed based on the method of Qi et al. (2014). The relative yield of melanin was measured with a spectrophotometer at 420 nm as described by Lü et al. (2017). The primers used are listed in Supplementary Table 8.

*ShSCD*-deletion mutants and wild-type *S. shiraiana* were inoculated in PDA medium containing 5 mM and 10 mM hydrogen peroxide, respectively. There were six plates in each group, and colony photography and diameter measurement were conducted after 9 days of culture. The experiments were repeated at least three times.

### Plant Treatment and Fungal Inoculation

Each group of ten 6-week-old *Arabidopsis* leaves were pretreated with CWDE solutions I (3.0% cellulase R-10), II (1.0% pectinase) or III (2.0% hemicellulase) for 30 h, respectively. The buffer used in the enzyme solution comprised 0.6 M D-mannitol, 20 mM potassium chloride, and 20 mM MES monohydrate (pH 5.7). Leaves pre-treated with this buffer served as negative controls. The pre-treated *Arabidopsis* leaves were washed with double-distilled water, and then excess water was removed with filter paper. The leaves were transferred to Petri dishes lined with moist filter paper and then inoculated with *S. shiraiana*. The leaves were photographed at 4 days after inoculation. The experiments were repeated at least three times.

### Accession Code

The *S. shiraiana* genome data have been deposited in the Genome Warehouse (GWH) in National Genomics Data Center, Beijing Institute of Genomics (BIG), Chinese Academy of Sciences, under accession number GWHACFG00000000 that is publicly accessible at https://bigd.big.ac.cn/gwh.

## Results

### Genome Sequencing and Assembly

**FIGURE 2:**
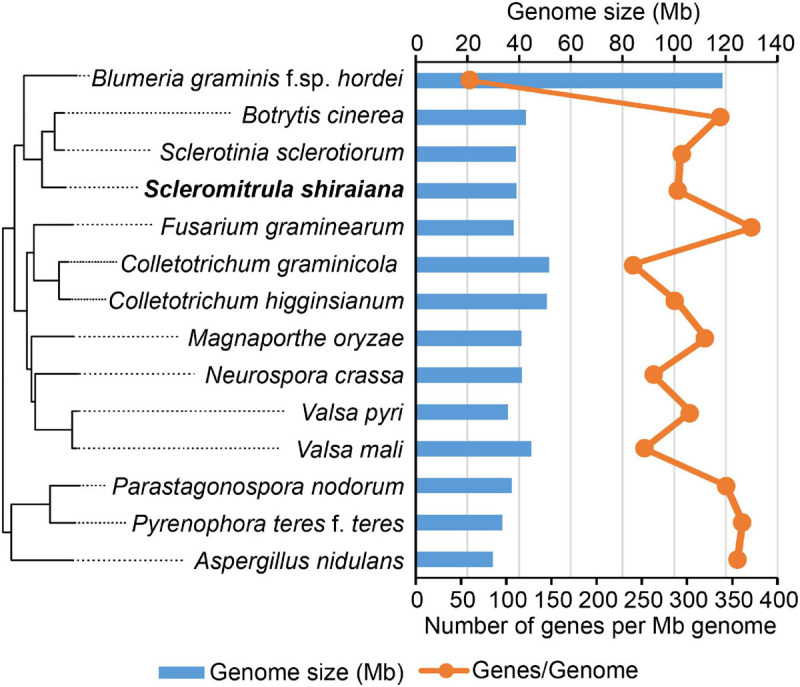
Genome size and the number of genes per mega genome sequence of *Scleromitrula shiraiana* and 13 other Ascomycetes.

To investigate the infection mechanism and to generate a high-quality reference genome for *S. shiraiana*, the genome of *S. shiraiana* strain SX-001 was sequenced by SMRT technology. A total of 3.40 Gb data were *de novo* assembled using SMRT Link v5.1.0. The genome sequence assembly contained 21 contigs with a total length of 39.0 Mb and a coverage of 87-fold (Table 1). The contig N50 was 3.61 Mb and the max-length contig was 4.96 Mb. The genome size and predicted gene number of *S. shiraiana* was similar to those of other related fungi, such as *S. sclerotiorum*, *B. cinerea*, and *Magnaporthe oryzae*, except for biotrophic *Blumeria graminis* f. sp. *hordei* (Figure 2 and Supplementary Table 1). However, the GC content in the genome sequence was lower in *S. shiraiana* (38.9%) than in other fungi (Supplementary Table 1).

**TABLE 1:** Genome features of *Scleromitrula shiraiana* strain SX-001.

**Features**	***S. shiraiana***
Assembly size (Mb)	39.0
Total reads (Gb)	3.4
Coverage	87×
Contig number	21
Max_length contig (Mb)	4.96
Contig N50 (Mb)	3.61
GC (%)	38.85
Predicted protein-coding genes	11327
Gene total length (Mb)	22.8
Gene average length (bp)	2012
Gene length/Genome (%)	58.4
Secreted proteins	884

**FIGURE 3:**
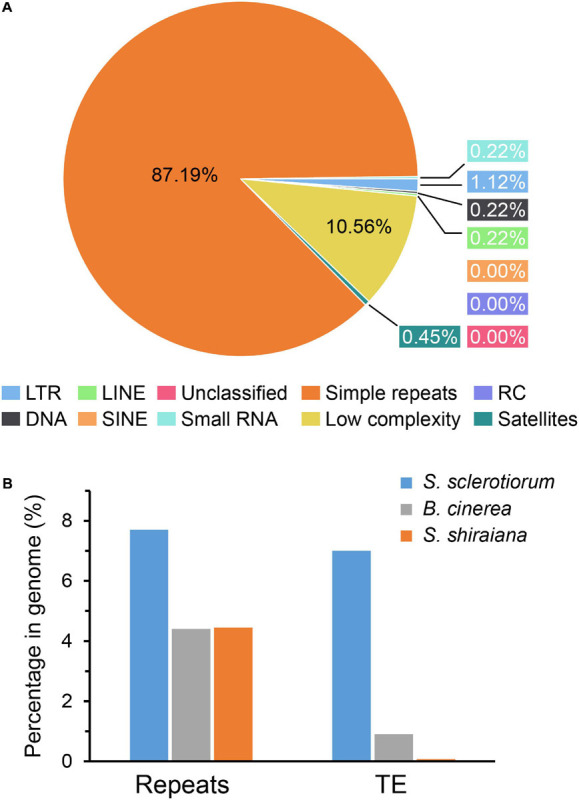
Repetitive sequences and transposable elements (TEs) in *Scleromitrula shiraiana* genome. **(A)** Repetitive sequences content of the genomes of *S. shiraiana* genome. **(B)** Comparison of the proportion of repeats and transposable elements among genomes of *Sclerotinia sclerotiorum*, *Botrytis cinerea*, and *S. shiraiana*. TEs include LTR, LINE, SINE, DNA, RC, and Unclassified. LTR, long terminal repeat; LINE, long interspersed nuclear element; SINE, short interspersed nuclear element; DNA, DNA transposon; RC, rolling circle; Unclassified, unclassified transposable elements.

The proportion of repetitive sequences in the genome of *S. shiraiana* (4.45%) was comparable to that in the genome of *B. cinerea* (4.4%), and lower than that in the genome of *S. sclerotiorum* (7.7%) (Figure 3). However, only 0.07% of the repetitive sequences were predicted to be transposable elements, which is significantly lower than that in *S. sclerotiorum* (7%) and in *B. cinerea* (0.9%) (Figure 3B).

### Mating Type (MAT) of *S. shiraiana*

**FIGURE 4:**
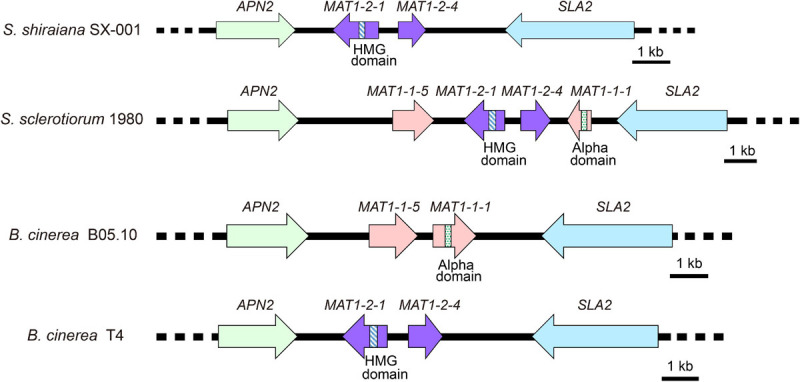
Comparative analysis of *MAT* loci among *Scleromitrula shiraiana* SX-001, *Sclerotinia sclerotiorum* 1980, and *Botrytis cinerea* (B05.10 and T4). Orthologous genes are shown with same color and pattern.

*Sclerotinia sclerotiorum* contains two types of *MAT* idiomorphs, *MAT1-1* and *MAT1-2*. The products encoded by *MAT1-1-1* and *MAT1-2-1* contain a conserved alpha domain and a high mobility group (HMG) domain, respectively. *B. cinerea* Strain B05.10 contains a *MAT1-1* idiomorph including a characteristic *MAT1-1-1* alpha-domain gene and *MAT1-1-5*. The *B. cinerea* strain T4 contains a *MAT1-2* idiomorph including a characteristic *MAT1-2-1* HMG-domain gene and *MAT1-2-4* (Faretra et al., 1988). The arrangement of the *MAT* locus of *S. shiraiana* SX-001 was similar to that of *B. cinerea* T4 (Figure 4). The *MAT1-2* idiomorph of *S. shiraiana* SX-001 contained *MAT1-2-1* and *MAT1-2-4*, but their encoded products showed only 37.0% and 29.1% similarity to their homologs in *B. cinerea* T4, respectively. These values were significantly lower than the similarity of MAT1 products between *B. cinerea* T4 and *S. sclerotiorum* (strain 1980), 77.0% and 65.1%, respectively (Supplementary Figure 1).

### Comparative Genomic Analysis

**FIGURE 5:**
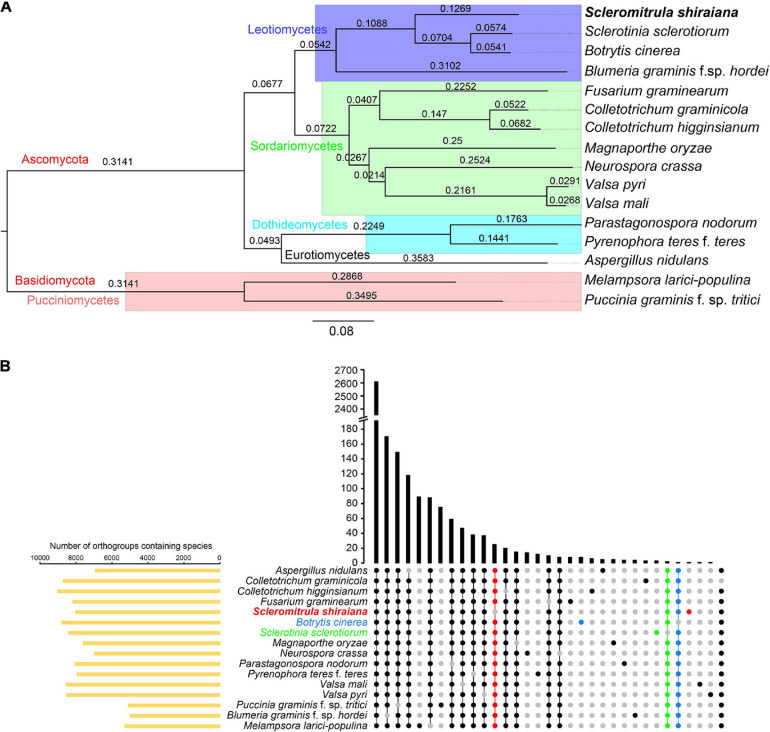
Phylogenetic and orthogroups analysis of *Scleromitrula shiraiana* and other fungal species. **(A)** Phylogenetic tree of different fungal species. Phylogenetic tree was constructed with STAG (Species Tree inference from All Genes) and STRIDE (Species Tree Root Inference from gene Duplication Events) methods with OrthoFinder2. *Melampsora larici-populina* and *Puccinia graminis* f. sp. *tritici* were used as outgroups. **(B)** Statistical analysis of Orthogroups of *S. shiraiana* and other 15 fungi. Orthogroups in each genome were identified using OrthoFinder with default parameters. Species-specific orthogroups and species-missing orthogroups in *S. shiraiana, Botrytis cinerea, Sclerotinia sclerotiorum* are highlighted.

The orthologous protein families were identified by OrthoFinder. The proteomes of *S. shiraiana* and 15 other fungi were clustered, and 15,558 ortholog families were obtained (Supplementary Tables 2,3). Of these ortholog families, 1266 single-copy orthologs were identified in all 16 fungi (Supplementary Table 3). In addition, there were 39,345 unassigned genes, accounting for 20.0% of the total number of genes. These 16 fungi shared 2,605 ortholog families and contained 157,162 genes (Figure 5B and Supplementary Table 3). A phylogenetic tree of these 16 fungi was constructed with STAG (Species Tree inference from All Genes) and STRIDE (Species Tree Root Inference from gene Duplication Events) methods (Figure 5A). In the phylogenetic tree, *S. shiraiana* was clustered with three other Leotiomycetes; *B. cinerea*, *S. sclerotiorum*, and *B. graminis* f. sp. *hordei*. Among the 16 fungi, *S. shiraiana* has more missing orthogroups than the closely related *S. sclerotiorum* and *B. cinerea* (Figure 5B).

Since *S. shiraiana*, *B. cinerea*, *S. sclerotiorum*, and *B. graminis* f. sp. *hordei* were clustered in the phylogenetic tree, and *B. graminis* f. sp. *hordei* is a special Leotiomycetes member (obligate biotroph, huge genomes, and few protein-coding genes), so *S. shiraiana*, *B. cinerea*, and *S. sclerotiorum* were analyzed separately. *S. shiraiana*, *B. cinerea*, and *S. sclerotiorum* belong to the same family (*Sclerotiniaceae*), but they are classified into three different genera. These three species shared 7,402 ortholog families (Supplementary Figure 2). Although *S. shiraiana* is evolutionally close to *B. cinerea* and *S. sclerotiorum*, genome coverage was only 20.9% and 21.4%, respectively (Supplementary Table 4). These values were lower than the coverage between *B. cinerea* and *S. sclerotiorum* (72.3%) (Amselem et al., 2011), and between *Colletotrichum higginsianum* and *Colletotrichum graminicola* (35.3%) (O’connell et al., 2012). These results suggest that *S. shiraiana* may have differentiated from its ancestors earlier than did *S. sclerotiorum* and *B. cinerea*.

### Oxalic Acid and Secondary Metabolism

Oxalic acid is an important pathogenic metabolite in *S. sclerotiorum* (Cessna et al., 2000; Kim et al., 2008; Williams et al., 2011). The enzyme OAH (oxaloacetate acetyl hydrolase) is associated with oxalic acid accumulation (Han et al., 2007; Liang et al., 2015; Xu et al., 2015). Oxalic acid is also produced by *S. shiraiana* (Lü et al., 2017). An *OAH* gene (Ssh_07163) was identified in the *S. shiraiana* genome. Further research is required to identify the key genes for oxalic acid biosynthesis and degradation and the role of oxalic acid in the pathogenicity of *S. shiraiana*.

**FIGURE 6:**
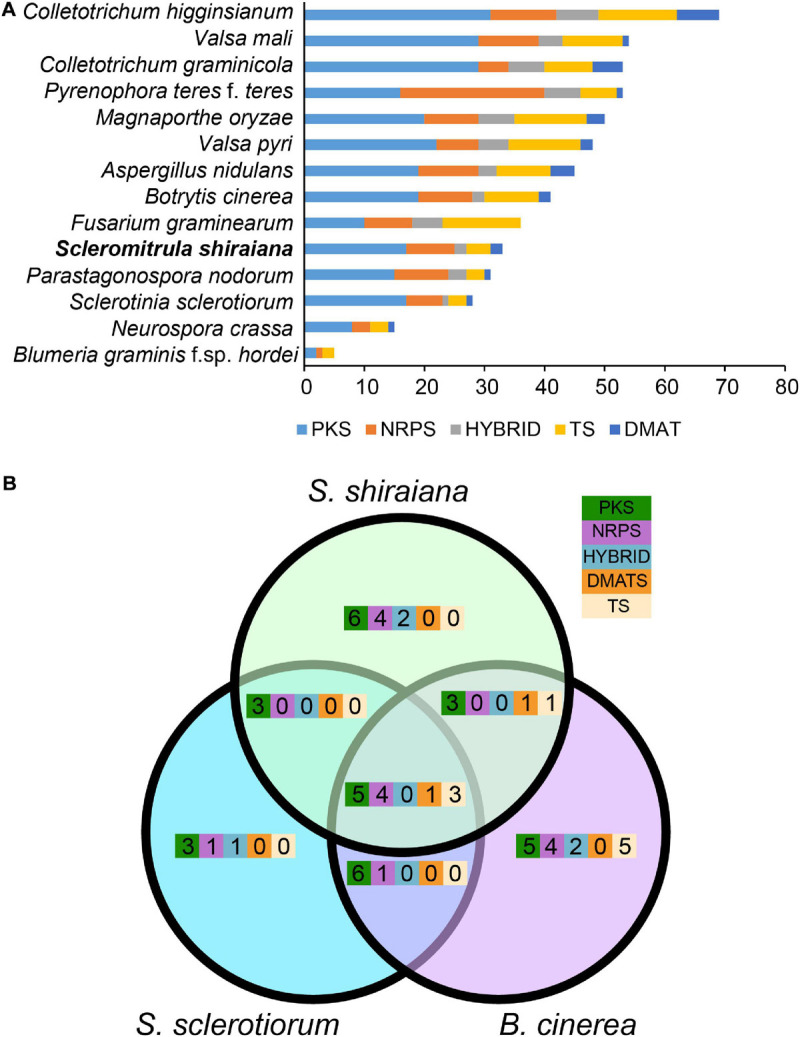
Comparison of secondary metabolism genes between *Scleromitrula shiraiana* and other Ascomycetes. **(A)** Comparison of genes encoding PKS, NRPS, HYBRID, TS, and DMATS between *S. shiraiana* and other 13 Ascomycetes. **(B)** Comparison of genes encoding PKS, NRPS, HYBRID, TS, and DMATS among *S. shiraiana, Botrytis cinerea*, and *Sclerotinia sclerotiorum.* PKS, NRPS, HYBRID, TS, and DMATS are represented by five different color rectangles. PKS, polyketide synthase; NRPS, non-ribosomal peptide synthase; HYBRID, hybrid NRPS–PKS enzyme; TS, terpene synthase; DMATS, dimethylallyl tryptophan synthase.

To identify the pathways involved in the production of secondary metabolites in *S. shiraiana*, a genome-wide search was conducted to identify genes encoding key enzymes such as PKS (polyketide synthase), NRPS (non-ribosomal peptide synthetase), HYBRID (hybrid NRPS–PKS enzyme), TS (terpene synthase), and DMATS (dimethylallyl tryptophan synthase). We detected 33 core biosynthetic genes encoding these five key enzymes, which were similar in number to those in *S. sclerotiorum*, but less than those in *B. cinerea* (Figure 6 and Supplementary Table 5). However, 36.3% of the core biosynthetic genes of *S. shiraiana* are species-specific and their function is unknown, indicating that *S. shiraiana* may produce species-specific secondary metabolites.

**FIGURE 7:**
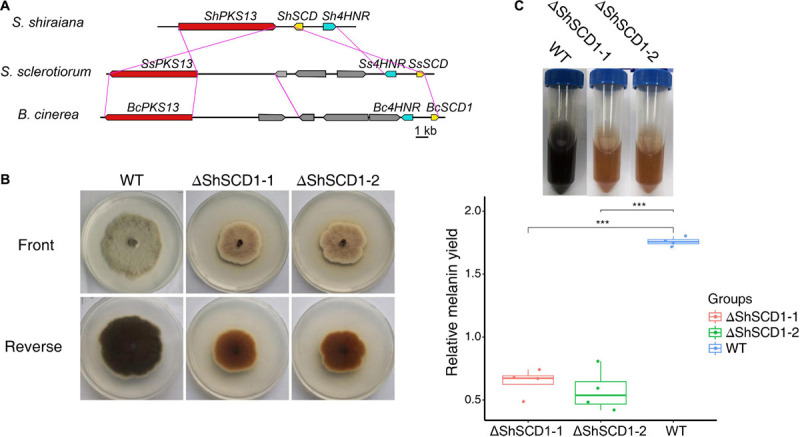
Cluster analysis of melanin biosynthetic genes in *Scleromitrula shiraiana* and functional analysis of *ShSCD*. **(A)** Comparison and analysis of melanin synthesis gene clusters among *S. shiraiana, Botrytis cinerea*, and *Sclerotinia sclerotiorum.* **(B)** Phenotypes of wild-type strain and *ShSCD-*deletion strains on potato dextrose agar (PDA) medium. **(C)**, Relative melanin yield in wild-type strain and *ShSCD*-deletion strains. Asterisks indicate a statistically significant difference (***p < 0.001) according to Student’s *t*-test (*n* = 4). Experiments were repeated at least three times.

Melanin is necessary for pathogen fitness (Henson et al., 1999; Liu and Nizet, 2009). The key genes for melanin synthesis in fungi are *PKS*, *SCD* (encoding scytalone dehydratase), and *4HNR* (encoding 1,3,6,8-tetrahydroxynaphthalene reductase). Homologs of these were found in *S. shiraiana*, and like in other fungi, they were arranged in the same gene cluster. However, the arrangement of these genes differed between *S. shiraiana* and *S. sclerotiorum/B. cinerea* (Figure 7A), suggesting that genomic rearrangement has occurred in *S. shiraiana*. To verify the effect of melanin on *S. shiraiana*, *ShSCD*-deletion mutants were obtained (Figure 7B and Supplementary Figure 4). Melanin production was significantly reduced in the *ShSCD*-deletion mutants (Figures 7B,C). Reactive oxygen species are related to plant defense against pathogens. This study showed that the ability of the *ShSCD*-deletion mutants to resist oxidative stress (H_2_O_2_) was lower than that of the wild type (Supplementary Figure 4). Unfortunately, pathogenicity assays have not been performed because the filamentous hyphae of *S. shiraiana* were difficult to inoculate mulberry, and *S. shiraiana* cannot form viable sclerotia in artificial medium (Lü et al., 2017). This may be related to the fact that the sclerotium of *S. shiraiana* is pseudosclerotium (Lü et al., 2017). Therefore, the pathogenicity of *ShSCD*-deletion mutants requires further determination.

### Reduced Number of Genes Encoding Plant CWDEs in *S. shiraiana*

The plant cell wall is an important barrier against pathogen attack (Kubicek et al., 2014). To overcome this barrier, phytopathogenic fungi produce a wide variety of secreted enzymes to degrade the major structural polysaccharide components of the plant cell wall. A comparative analysis showed that there were fewer genes encoding carbohydrate-active enzymes (CAZymes) in *S. shiraiana* than in the other hemibiotrophic and necrotrophic fungi being analyzed. The comparison also showed that the number of CAZymes in necrotrophic pathogenic fungi was similar or slightly less than that in the hemibiotrophic pathogenic fungi, but more than that in biotrophic and saprophytic fungi. However, there were significantly fewer CAZymes in *S. shiraiana* than those of restricted host range necrotrophic *P. nodorum* and *P. teres* f. *teres* (Table 2).

**TABLE 2:** Comparison of carbohydrate-active enzyme (CAZyme) classes from *S. shiraiana* and 15 other fungal genomes.

**Nutrition mode**	**Species**	**GH**	**GT**	**PL**	**CE**	**AA**	**CBM**
Hemibiotroph	*Colletotrichum higginsianum*	323	108	48	114	157	20
	*Colletotrichum graminicola*	278	91	17	89	130	16
	*Fusarium graminearum*	250	97	22	87	102	17
	*Magnaporthe oryzae*	249	93	6	82	115	14
Necrotroph	*Botrytis cinerea*	270	106	10	82	109	8
	*Valsa mali*	265	88	13	63	105	9
	*Valsa pyri*	260	86	14	63	100	9
	*Parastagonospora nodorum*	251	76	10	47	127	11
	*Pyrenophora teres* f. *teres*	236	87	10	45	115	8
	*Sclerotinia sclerotiorum*	218	84	5	63	79	15
	***Scleromitrula shiraiana***	**197**	**79**	**4**	**32**	**91**	**10**
Saprotroph	*Aspergillus nidulans*	254	79	23	66	85	20
	*Neurospora crassa*	189	85	4	44	69	13
Obligate biotroph	*Melampsora larici-populina*	168	70	8	57	47	1
	*Puccinia graminis* f. sp. *tritici*	150	80	3	43	28	1
	*Blumeria graminis* f. sp. *hordei*	54	54	0	20	11	2

*GH, Glycoside hydrolase family; GT, Glycosyltransferase family; PL, Polysaccharide lyase family; CE, Carbohydrate esterase family; CBM, Carbohydrate-binding module family; AA, Auxiliary activities family.*

**FIGURE 8:**
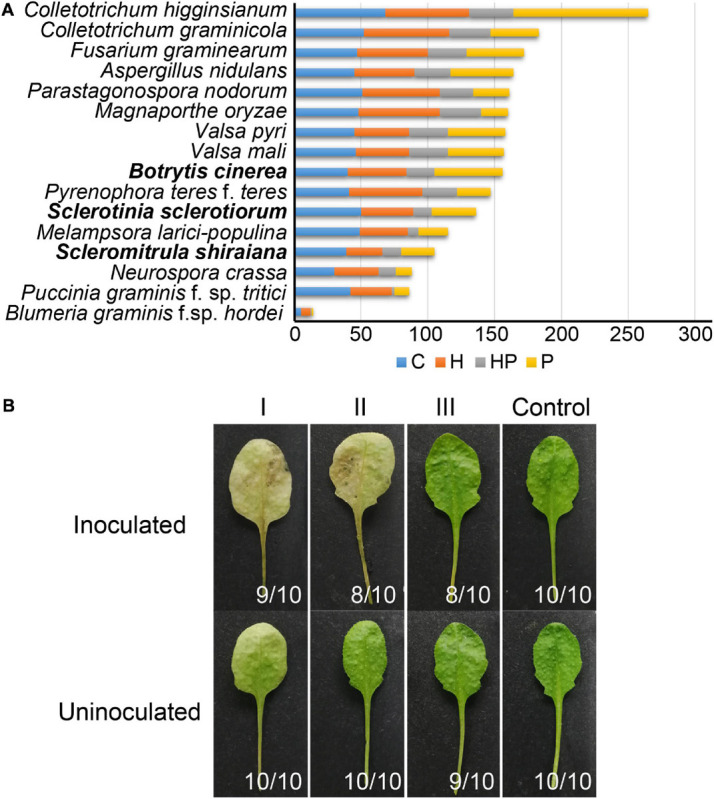
Genes encoding plant cell wall-degrading enzymes (CWDEs) in *Scleromitrula shiraiana*. **(A)** Comparison of genes encoding CWDEs between *S. shiraiana* and 15 other fungi. C, cellulase; H, hemicellulase; HP, hemicellulase or pectinase (degrade hemicellulose and pectin side chains); P, pectinase. **(B)** Effects of additional application of cell wall-degrading enzymes on pathogenicity of *S. shiraiana* against *Arabidopsis*. *Arabidopsis* leaves were pretreated buffer (negative control) or CWDE solution I (3.0% cellulase R-10), II (1.0% pectinase), or III (2.0% hemicellulase) for 30 h, washed with double-distilled water, inoculated with *S. shiraiana*, and photographed at 4 days after inoculation. The number in the lower right corner of each leaf indicates the number of leaves with the corresponding phenotype/number of all detected leaves. The experiments were repeated at least three times.

The number of genes encoding CWDEs of *S. shiraiana* was 67.3% of that of *B. cinerea* and 77.2% of that of *S. sclerotiorum* (Supplementary Table 6). Among all the necrotrophic pathogens compared, *S. shiraiana* had the smallest number of genes encoding CWDEs (Figure 8A and Supplementary Table 6). Some Ascomycetes, such as *S. sclerotiorum* and *B. cinerea*, *Valsa mali*, and *Valsa pyri*, are suited for pectin decomposition. However, *S. shiraiana* had significantly fewer genes encoding polygalacturonases and rhamnogalacturonases (GH28), and pectate lyase and pectin lyase (PL1), which are necessary for efficient degradation of pectin (Figure 8A and Supplementary Table 6). The number of genes encoding hemicellulases also differed widely among the 16 fungal genomes. Among Ascomycetes, there is only one gene encoding acetyl xylan esterase (CE1) and endo-β-1,4-xylanase (GH11) in *S. shiraiana*, while other fungi contain two or more (Supplementary Table 6). Acetylesterases (CE16) are also less abundant in *S. shiraiana* than in *S. sclerotiorum* and *B. cinerea*. In addition, β-mannanase (GH26) is absent in *S. shiraiana*, as well as *P. nodorum* and *P. teres* f. *teres*, *B. graminis* f. sp. *hordei*, *Fusarium graminearum*, and *M. oryzae*. Interestingly, with the exception of *S. shiraiana*, the other five fungi are restricted or obligate infecting gramineous plants.

We hypothesized that the small number of genes encoding CWDEs may explain the weak pathogenicity of *S. shiraiana*. To test this hypothesis, we conducted experiments using *Arabidopsis* leaves insensitive to *S. shiraiana*. The *Arabidopsis* leaves were pretreated with cellulase, pectinase, and hemicellulase, and then inoculated with *S. shiraiana*. The application of cellulase and pectinase allowed *S. shiraiana* to infect *Arabidopsis* leaves, but the application of hemicellulase did not (Figure 8B). This result suggests that the small number of CWDEs in *S. shiraiana* may be involved in its limited host range.

### Functional Analysis of Putative Effectors

**FIGURE 9:**
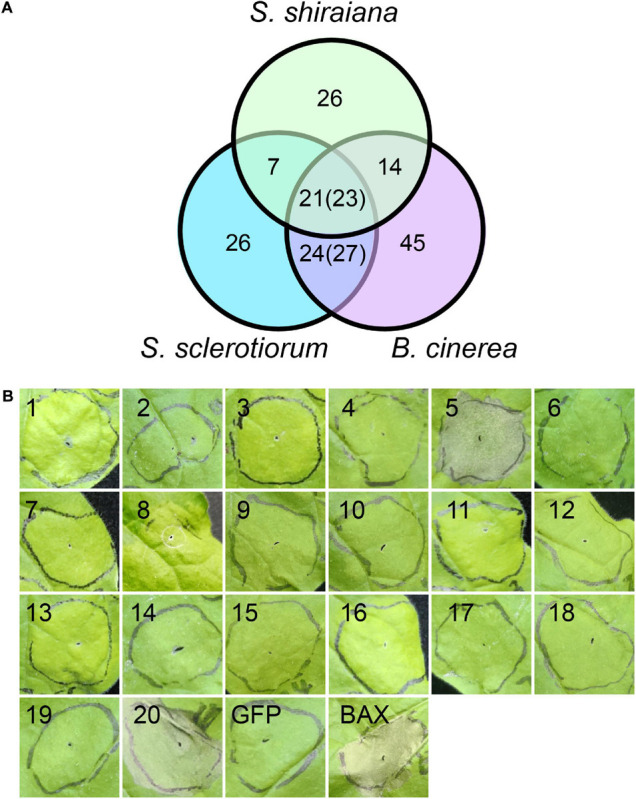
Prediction and analysis of effector proteins in *Scleromitrula shiraiana*. **(A)** Comparison of effectors predicted in *S. shiraiana, Sclerotinia sclerotiorum*, and *Botrytis cinerea.* Numbers in parentheses indicate that multiple effector proteins in *B. cinerea* are shared with *S. sclerotiorum* and/or *S. shiraiana*. **(B)** Identification of putative *S. shiraiana* effectors that can induce cell death in *Nicotiana benthamiana.* Leaves of 4–6-week-old *N. benthamiana* plants were infiltrated with *Agrobacterium* carrying *S. shiraiana* effector genes using a 1-ml needleless syringe (positive control, BAX; negative control, GFP). Cell death symptoms were photographed at 6 days after infiltration. Results are representative of three biological replicates.

Plant pathogens secrete effector proteins to suppress plant defense responses and modulate host cellular processes to promote colonization (Lo Presti and Kahmann, 2017). The function of most fungal effector proteins is unknown, and most of them lack conserved domains or homologs in other species. We detected 68 genes encoding putative effector proteins in *S. shiraiana*, which was less than 78 in *S. sclerotiorum* and 109 in *B. cinerea* (Figure 9A). As expected, most of the 68 predicted effector proteins lacked functional or conserved domains. Moreover, there are 26 effector proteins that were species-specific (Supplementary Table 7). In order to initially determine which ones cause host cell death, 20 effector protein genes were randomly selected and transiently expressed in *Nicotiana benthamiana* by *Agrobacterium* infiltration. The effectors sshi00003413 (E5) and sshi00010565 (E20) strongly induced cell death (Figure 9B and Supplementary Figure 5), suggesting that these two effector proteins may also cause cell death in the mulberry, thereby promoting the colonization and infection of pathogen. Both effector proteins have no conserved domains.

## Discussion

Mulberry sclerotial disease is the most destructive fungal disease of mulberry, and often causes high yield losses. *S. shiraiana* is one of the causal pathogens of mulberry sclerotial disease, and is also the one that is easiest to isolate and cultivate artificially. In addition, *S. shiraiana* is a necrotrophic pathogenic fungus with a restricted host range; it is only found on mulberry. In this study, we sequenced the genome of *S. shiraiana* and obtained a high-quality 39.0 Mb assembly with 21 contigs. The genome size and predicted gene number of *S. shiraiana* is similar to that of most other Ascomycetes, including the taxonomically closely related *S. sclerotiorum* and *B. cinerea*. *S. shiraiana*, *S. sclerotiorum*, and *B. cinerea* belong to the *Sclerotiniaceae*, but *S. shiraiana* may have emerged earlier from their common ancestor species. Navaud et al. (2018) divided *Sclerotiniaceae* into three major macro-evolutionary regimes according to the variation of diversification rate during the evolution, which have contrasted proportions of broad host range species. The fungi of the regime G1 including *S. shiraiana* are early diverging, and showed low diversification rates and low host jump frequencies resulted in a narrow host range (Navaud et al., 2018). The emergence of broad host range fungi (such as *S. sclerotiorum* in regime G2 and *B. cinerea* in regime G3) is largely driven by high jump frequency and probably combined with low diversification rate (Navaud et al., 2018). *S. shiraiana* shares some characteristics with *S. sclerotiorum* and *B. cinerea*. First, *S. shiraiana* and *B. cinerea* are heterothallic, while *S. sclerotiorum* is homothallic. Second, the sexual stages of *S. shiraiana* and *S. sclerotiorum* are critical to their life cycle, and the ascospores produced in their sexual process are the main primary infection source. However, the sexual stage of *B. cinerea* is very rare in nature (Dewey and Grant-Downton, 2016). Third, *S. shiraiana* and *B. cinerea* can produce conidiospores in the asexual stage, while *S. sclerotiorum* cannot (Saharan and Mehta, 2008). Fourth, in terms of genome composition, there are more transposable elements in the *S. sclerotiorum* genome than in the genomes of *S. shiraiana* and *B. cinerea*. Of course, *S. shiraiana* also has many differences from *S. sclerotiorum* and *B. cinerea*. First and foremost, *S. shiraiana* has a narrow host range, and it is likely to infect only *Morus* plants. The adaptation of *S. shiraiana* to mulberry restricted its infection to other potential hosts. In addition, the hyphae of *S. shiraiana* grow very slowly and easily produce a large amount of melanin in artificial culture medium. This suggests that *S. shiraiana* is weak in competitiveness and environmental adaptability. Another difference is that, *B. cinerea* depends on its vigorous conidia to infect plants. Although *S. shiraiana* also produces conidia, their role in its life cycle is dispensable or negligible. One of the reasons is that the mulberry flowering period is short and a very narrow time window is available for conidia reinfection. The other and the main reason is that the conidia of *S. shiraiana* are produced in a liquid environment, which severely limits its spread (Lü et al., 2017). This may also be one of the reasons that restrict *S. shiraiana* from jumping to and infecting other potential host plants.

The plant cell wall is a vital defense barrier and a main component of the defense monitoring system (Bacete et al., 2018). The plant cell wall is a heterogeneous structure composed of polysaccharides, proteins, and aromatic polymers. The main components of polysaccharides are cellulose, hemicellulose, and pectin. Degradation of plant cell wall polysaccharides is an important infection strategy for necrotrophs (Glass et al., 2013). In addition, degraded polysaccharides serve as their carbon sources. As shown in the comparative genomic analysis, there are fewer genes encoding carbohydrate-active enzymes in the *S. shiraiana* genome than in the genomes of other necrotrophs. Cellulase, hemicellulase, and pectinase target cellulose, hemicellulose, and pectin in the plant cell wall network complex, respectively. The number of genes encoding CWDEs in the *S. shiraiana* genome is significantly less than that of *S. sclerotiorum*, *B. cinerea* and restricted host range necrotrophic *P. nodorum* and *P. teres* f. *teres*. In fact, except for *B. cinerea*, most other *Botrytis* spp. have a narrow host range. The difference in the number of CWDEs of *Botrytis* spp. is closely related to the content of their host cell wall components (Valero-Jiménez et al., 2019). The initial colonization of *S. shiraiana* started from the stigma of decayed mulberry female flowers. The adaptation to mulberry allows *S. shiraiana* to successfully infect the host with less CWDEs. Our results show that cellulase and pectinase, rather than hemicellulase, significantly promote the infection of *A. thaliana* by *S. shiraiana*. However, this experiment is preliminary and has limitations. Because CWDEs pretreatment can enhance the susceptibility of plants to many microorganisms. So, we have not been able to determine the role of cellulase, pectinase, and even hemicellulase in the process of *S. shiraiana* infecting mulberries. The role of CWDEs in the colonization and pathogenicity of *S. shiraiana* needs further study.

Many plant pathogens, including bacteria, fungi, and oomycetes, secrete effector proteins that function in the apoplast or cytoplasm of host cells to suppress the immune response of plants (Macho and Zipfel, 2015; Toruno et al., 2016; Wang et al., 2019). Some isolates of *M. oryzae* can infect *Oryza sativa* japonica varieties because of their abundant effector proteins (Liao et al., 2016). We detected 68 predicted effector proteins in *S. shiraiana*, fewer than in *S. sclerotiorum*, *B. cinerea*, and other pathogens (Schirawski et al., 2010; O’connell et al., 2012; Yin et al., 2015; Derbyshire et al., 2017). The small number of CWDEs and effector proteins in *S. shiraiana* may be the one of the main reasons for its weak infectivity, which results in a narrow host range. However, some *Botrytis* spp. with a narrow host range and some regionally diverged isolates (such as *S. sclerotiorum* and *B. cinerea*) have specific effectors or abnormal numbers of CWDEs, which may have potentially undergone some level of host-specific adaptation (Mousavi-Derazmahalleh et al., 2019; Valero-Jiménez et al., 2019). Some reports indicate that *S. sclerotiorum* and other suspected pathogens are present on diseased mulberry fruit, but it is unknown whether these fungi cause mulberry sclerotial disease. Because *S. sclerotiorum* and *B. cinerea* often cause the rot of infected tissues, this is not conducive to the formation of their sclerotia in mulberries.

The comparative genomic analysis showed that one third of the genes encoding secondary metabolism core enzymes in *S. shiraiana* were species-specific. Different necrotrophic fungi secrete different types of phytotoxins to suppress the host immune response and promote disease. Botrydial produced by *B. cinerea* induces a hypersensitive response in the host plant, which contributes to pathogen infection (Rossi et al., 2011). ToxA, a host-specific toxin produced by *Pyrenophora tritici-repentis* and *Stagonospora nodorum*, enhances virulence against wheat (Faris et al., 2010). As a pathogen with a narrow host range, *S. shiraiana* may produce certain phytotoxins that make it more suitable for infecting mulberry.

In this study, we sequenced the necrotrophic pathogen that causes mulberry sclerotial disease, *S. shiraiana*. Usually, necrotrophic phytopathogenic fungi deploy phytotoxins, CWDEs, and effector proteins to cause plant cell necrosis and infect plants. However, the genome of *S. shiraiana* encodes far fewer CWDEs and effector proteins than do the genomes of closely related species. This may be one reason for the restricted host range of *S. shiraiana*. The differences in secondary metabolism genes between *S. shiraiana* and other Ascomycetes may be responsible its narrow host range. Although *S. shiraiana* has a small arsenal, it retains highly targeted virulence weapons for pathogenicity against mulberry.

## Data Availability Statement

The datasets presented in this study can be found in online repositories. The names of the repository/repositories and accession number(s) can be found below: https://bigd.big.ac.cn/gwh, GWHACFG00000000.

## Author Contributions

NH and ZL contributed to the conception and design of the work. ZL, ZH, LH, XK, and HL performed the experiments. ZH performed effector screening. ZL and LH performed cell wall degrading enzymes detection. XK performed *ShSCD* functional analysis. ZL, BM, YL, and JY analyzed the data. YL and JY contributed reagents and plant materials. ZL and ZH wrote the manuscript. NH, ZL, and BM produced the final version of the manuscript. All authors contributed to the article and approved the submitted version.

## Conflict of Interest

The authors declare that the research was conducted in the absence of any commercial or financial relationships that could be construed as a potential conflict of interest.

## Footnotes

1 https://mycocosm.jgi.doe.gov/mycocosm/home

2 http://fungi.ensembl.org/index.html

## Supplementary Material

Supplementary Figure 1 Sequence alignment of products encoded by genes at *MAT1-2* loci of *Scleromitrula shiraiana* SX-001, *Sclerotinia sclerotiorum* 1980, and *Botrytis cinerea* T4. **(A,B)**, Sequence alignment of MAT1-2-1 and MAT1-2-4 in *S. shiraiana* SX-001, *S. sclerotiorum* 1980 and *B. cinerea* T4, respectively. Sequence identity of SshMAT1-2-1 vs. BcMAT1-2-1, SshMAT1-2-1 vs. SscMAT1-2-1 and SscMAT1-2-1 vs. BcMAT1-2-1 is 37.0%, 41.6%, and 77.0%, respectively. Sequence identity of SshMAT1-2-4 vs. BcMAT1-2-4, SshMAT1-2-4 vs. SscMAT1-2-4 and SscMAT1-2-4 vs. BcMAT1-2-4 is 29.1%, 32.1% and 65.1%, respectively. Ssh, *S. shiraiana* SX-001; Bc, *B. cinerea* T4; Ssc, *S. sclerotiorum* 1980.

Supplementary Figure 2 Statistical analysis of orthogroups in *Scleromitrula shiraiana* SX-001, *Sclerotinia sclerotiorum* 1980, and *Botrytis cinerea* B05.10.

Supplementary Figure 3 Southern blotting verification of *ShSCD-*deletion strains.

Supplementary Figure 4 The tolerance test of *ShSCD*-deletion mutant to hydrogen peroxide. **(A)** High concentrations of hydrogen peroxide significantly inhibit the growth of *ShSCD*-deletion mutants. The colony inhibition rate = | D_*T*_ - D_*C*_| /D_*C*_. D_*T*_, the colony diameter of treatment group. D_*C*_, the colony diameter of control group. Different letters (a and b) indicate statistical differences (*p* < 0.05). **(B)** The colony morphology of *ShSCD*-deletion mutants and wild-type strain on PDA medium containing different concentrations of hydrogen peroxide. Colony diameter measurement and colony photography were performed after 9 days of culture. The experiments were repeated at least three times.

Supplementary Figure 5 The relative expression level of 20 effector genes transiently expressed in *N. benthamiana.*

Supplementary Table 1 Information for fungal genome used in this study.

Supplementary Table 2 Orthogroups gene count in 16 fungi.

Supplementary Table 3 Statistics of orthogroups and genes per species.

Supplementary Table 4 Coverage of *Scleromitrula shiraiana* sequences to those of *Sclerotinia sclerotiorum* and *Botrytis cinerea.*

Supplementary Table 5 Secondary metabolism key enzymes encoding genes in *Scleromitrula shiraiana*, *Sclerotinia sclerotiorum*, and *Botrytis cinerea.*

Supplementary Table 6 Comparison of plant and fungal cell wall-degrading enzymes between *Scleromitrula shiraiana* and 15 other fungi.

Supplementary Table 7 Protein domain analysis of 68 putative effectors of *Scleromitrula shiraiana.*

Supplementary Table 8 Primers used in this study.
