# Supplementary figures and images for: Genome Sequencing Analysis of Scleromitrula shiraiana, a Causal Agent of Mulberry Sclerotial Disease With Narrow Host Range
Source: Front Microbiol. 2021 Jan 14;11:603927. doi: 10.3389/fmicb.2020.603927 (PMC7840784; doi:10.3389/fmicb.2020.603927)

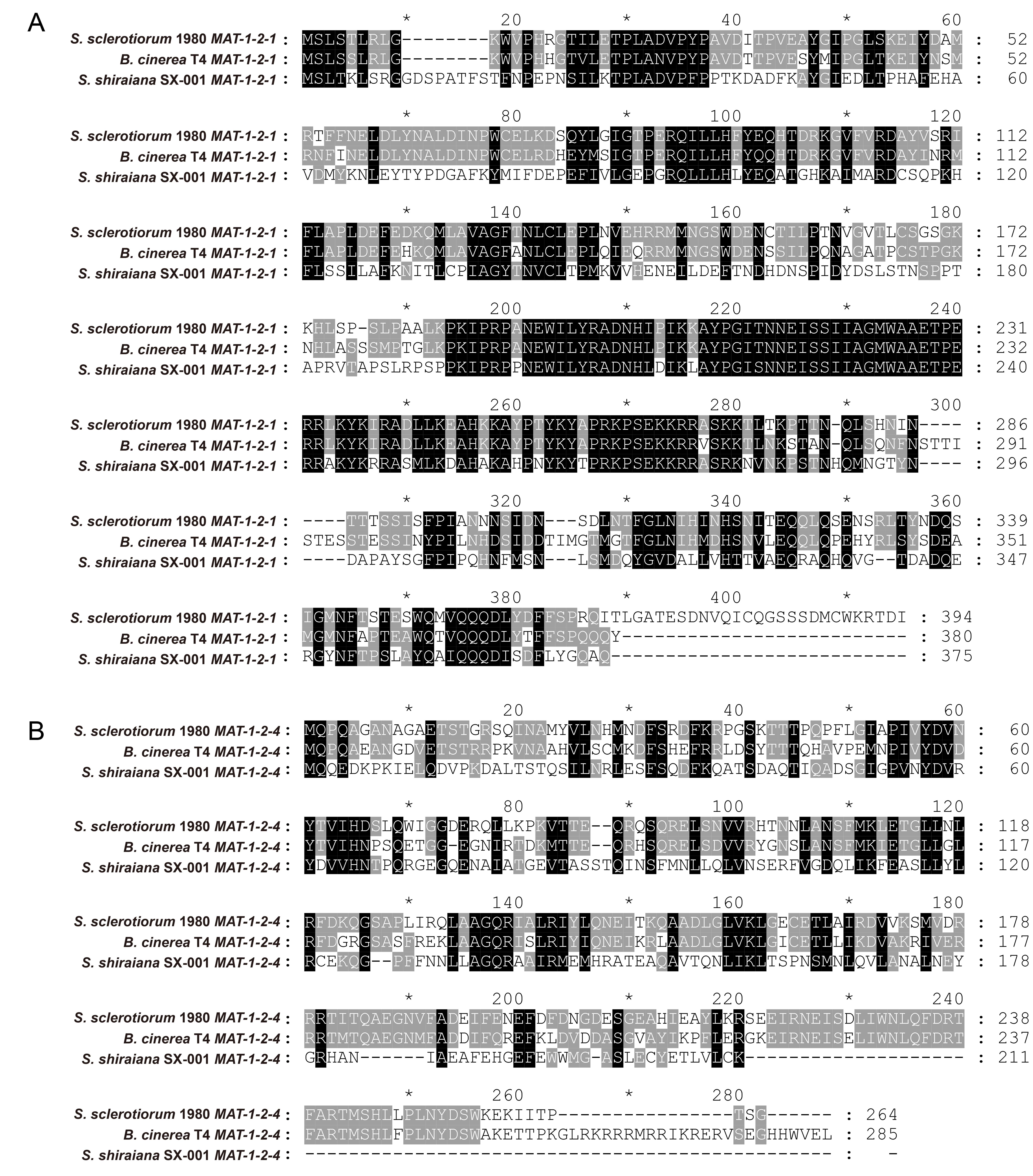

Supplement: Supplementary Figure 1 — Sequence alignment of products encoded by genes at MAT1-2 loci of Scleromitrula shiraiana SX-001, Sclerotinia sclerotiorum 1980, and Botrytis cinerea T4. (A,B), Sequence alignment of MAT1-2-1 and MAT1-2-4 in S. shiraiana SX-001, S. sclerotiorum 1980 and B. cinerea T4, respectively. Sequence identity of SshMAT1-2-1 vs. BcMAT1-2-1, SshMAT1-2-1 vs. SscMAT1-2-1 and SscMAT1-2-1 vs. BcMAT1-2-1 is 37.0%, 41.6%, and 77.0%, respectively. Sequence identity of SshMAT1-2-4 vs. BcMAT1-2-4, SshMAT1-2-4 vs. SscMAT1-2-4 and SscMAT1-2-4 vs. BcMAT1-2-4 is 29.1%, 32.1% and 65.1%, respectively. Ssh, S. shiraiana SX-001; Bc, B. cinerea T4; Ssc, S. sclerotiorum 1980. [file Image_1.TIF]

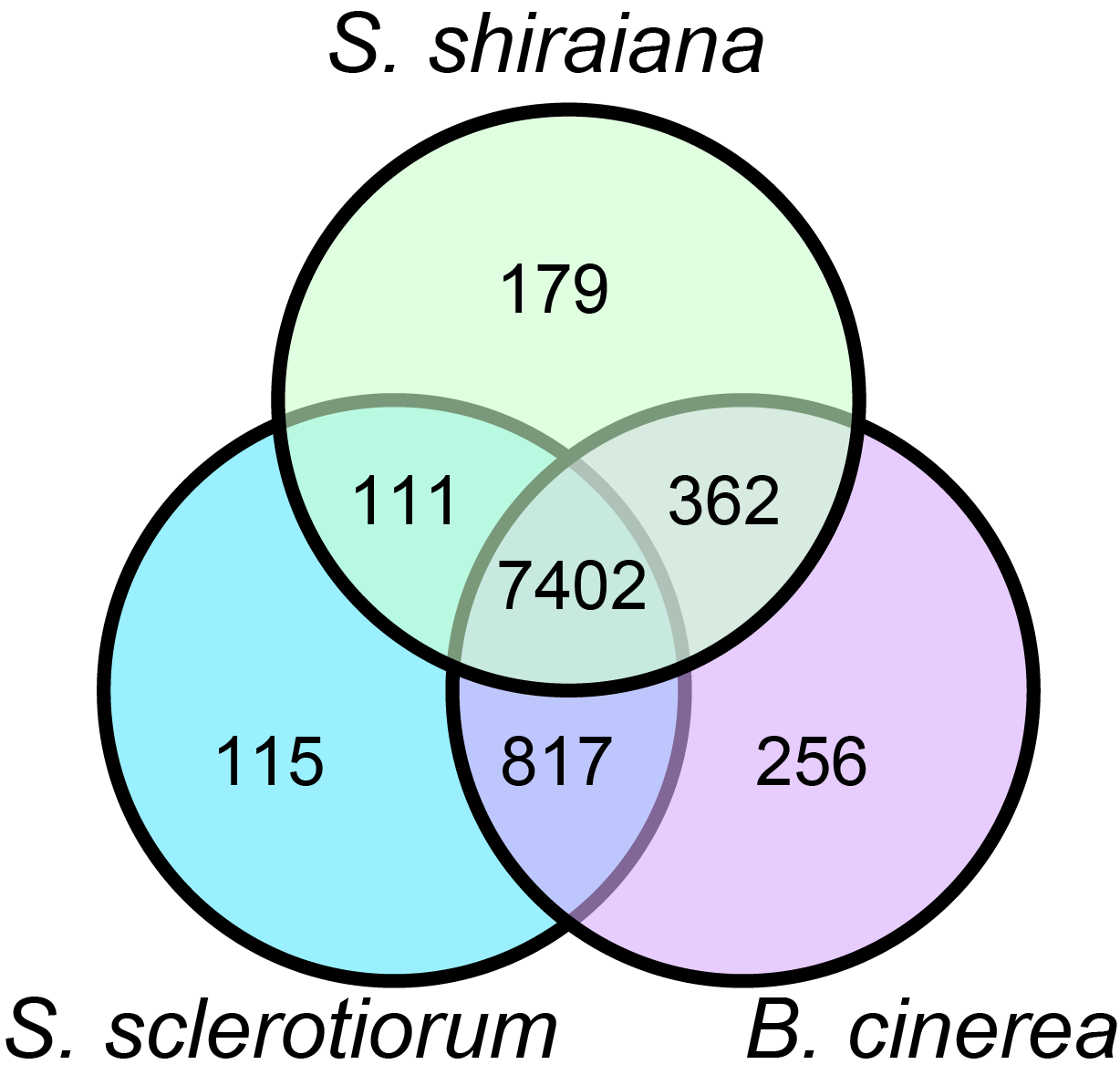

Supplement: Supplementary Figure 2 — Statistical analysis of orthogroups in Scleromitrula shiraiana SX-001, Sclerotinia sclerotiorum 1980, and Botrytis cinerea B05.10. [file Image_2.TIF]

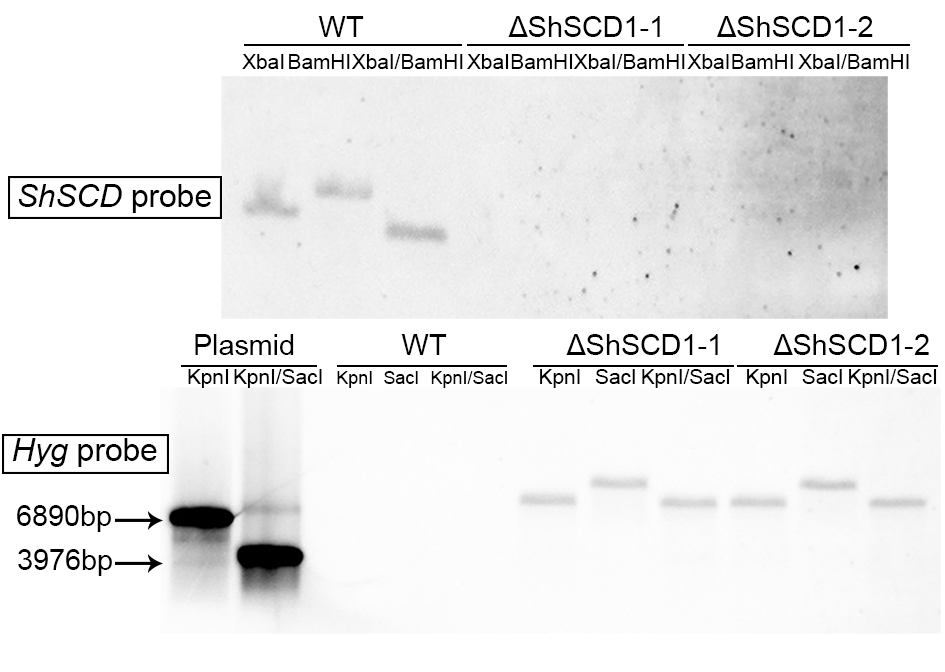

Supplement: Supplementary Figure 3 — Southern blotting verification of ShSCD-deletion strains. [file Image_3.TIF]

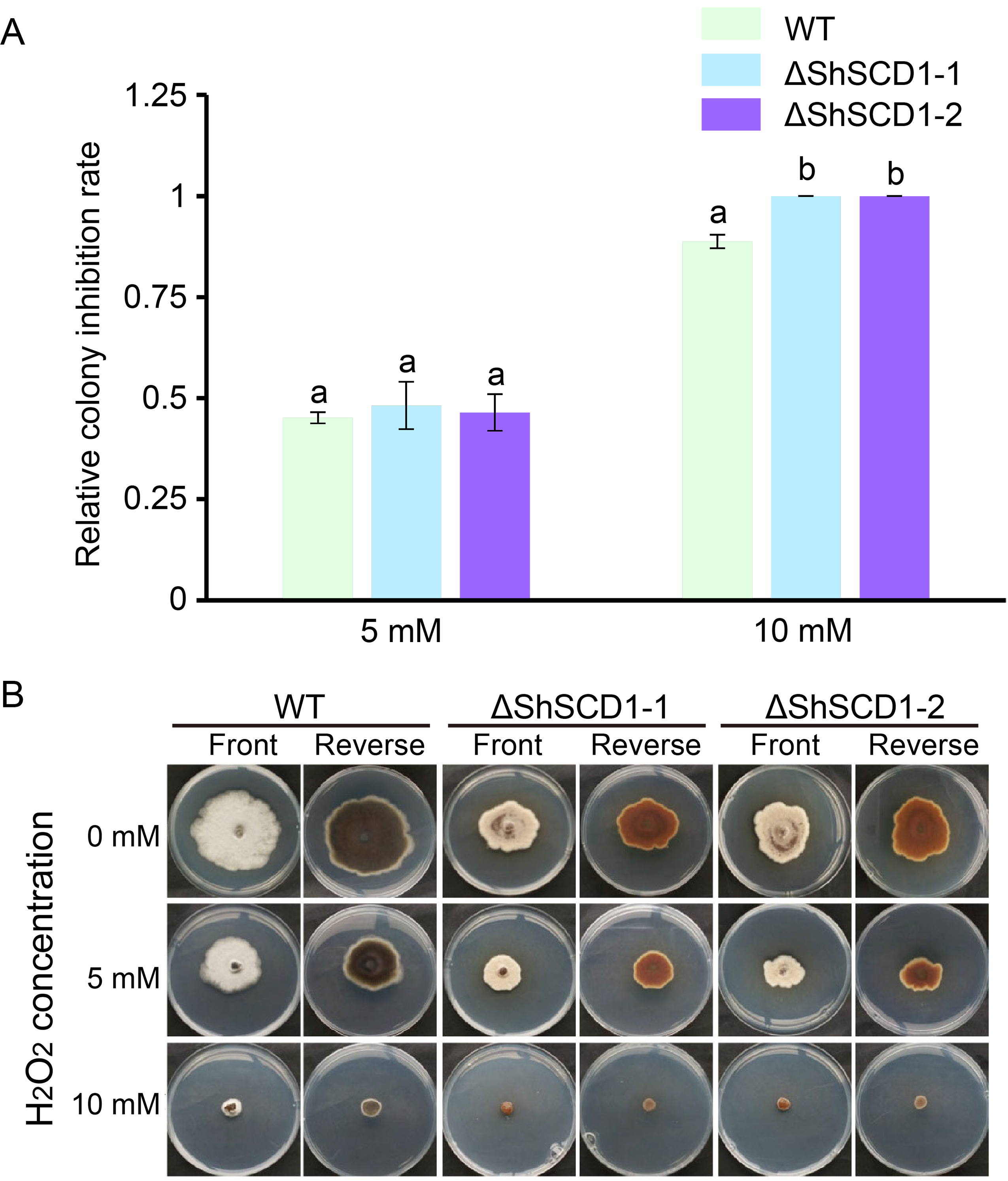

Supplement: Supplementary Figure 4 — The tolerance test of ShSCD-deletion mutant to hydrogen peroxide. (A) High concentrations of hydrogen peroxide significantly inhibit the growth of ShSCD-deletion mutants. The colony inhibition rate = | DT - DC| /DC. DT, the colony diameter of treatment group. DC, the colony diameter of control group. Different letters (a and b) indicate statistical differences (p < 0.05). (B) The colony morphology of ShSCD-deletion mutants and wild-type strain on PDA medium containing different concentrations of hydrogen peroxide. Colony diameter measurement and colony photography were performed after 9 days of culture. The experiments were repeated at least three times. [file Image_4.TIF]

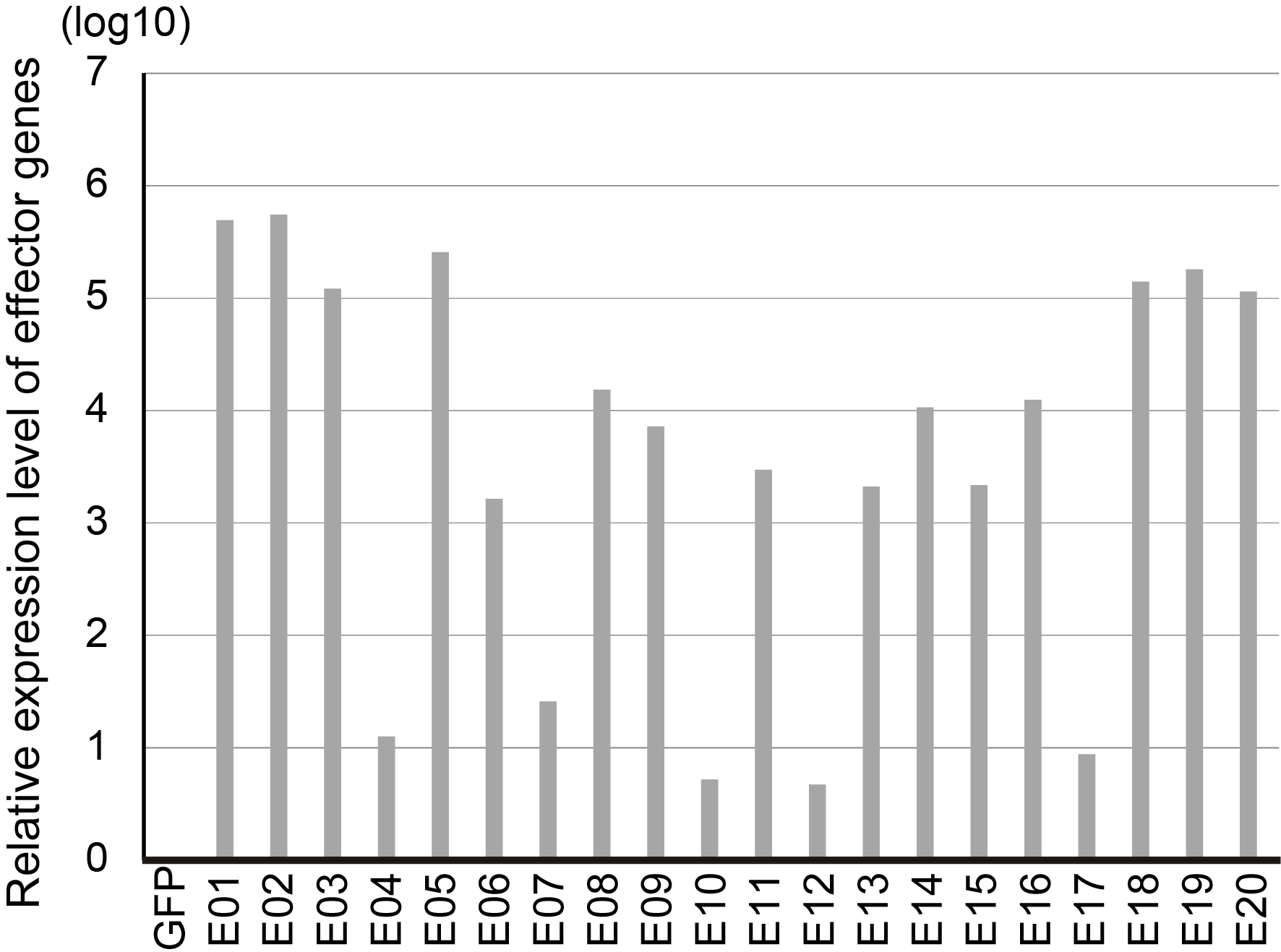

Supplement: Supplementary Figure 5 — The relative expression level of 20 effector genes transiently expressed in N. benthamiana. [file Image_5.TIF]
